# Supplementary material for: Hepatic Crtc2 controls whole body energy metabolism via a miR-34a-Fgf21 axis
Source: Nat Commun. 2017 Nov 30;8:1878. doi: 10.1038/s41467-017-01878-6 (PMC5709393; doi:10.1038/s41467-017-01878-6)
Supplement: Supplementary file 1 — Supplementary Information [file 41467_2017_1878_MOESM1_ESM.docx]

**Supplementary Information**


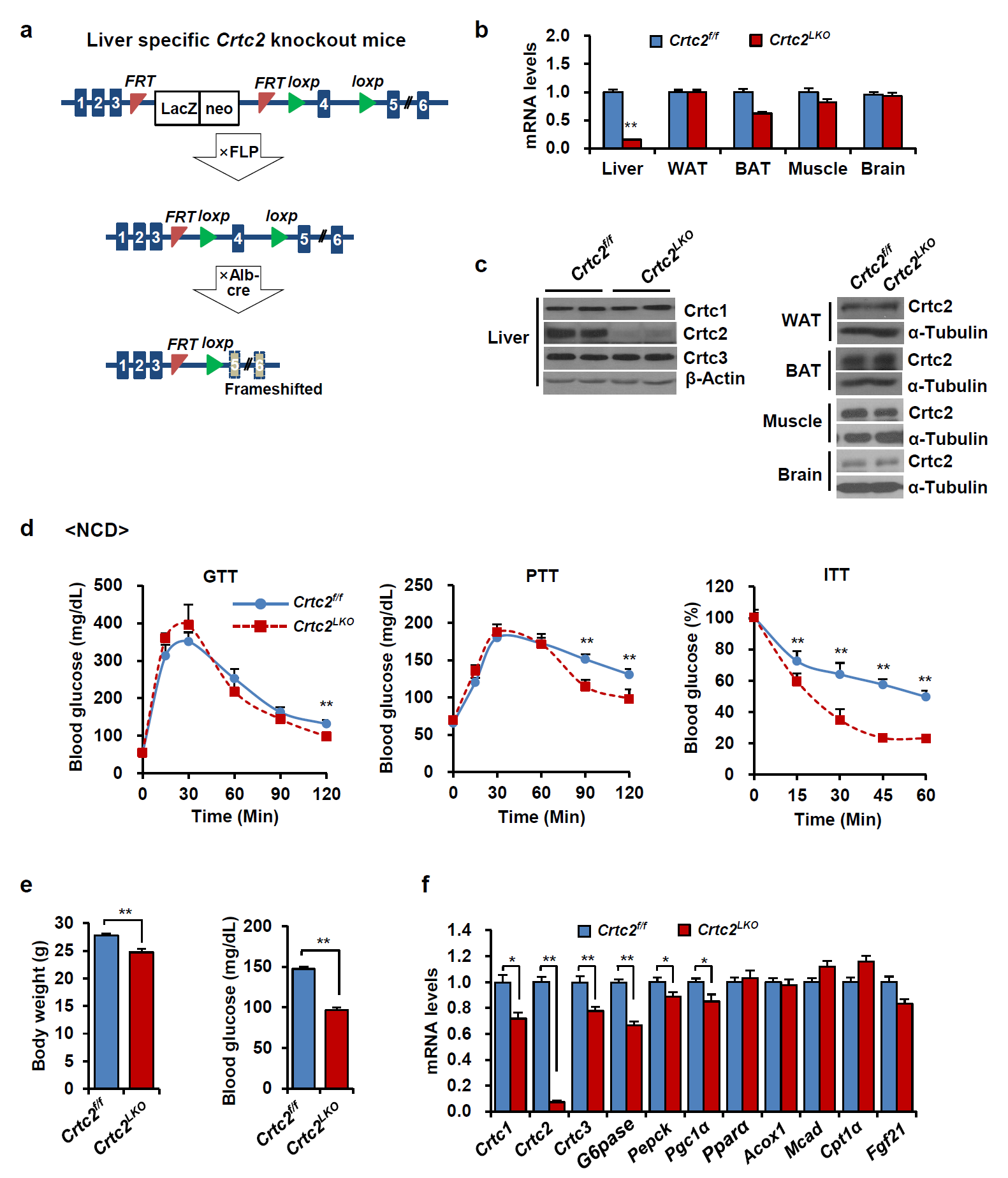


**Supplementary Figure 1. Generation of liver-specific *Crtc2* knockout mice.**

**a.** Schematic diagram showing the targeting strategy for generating liver-specific *Crtc2* knockout mice. **b**-**c.** Confirmation of liver-specific depletion of *Crtc2* in *Crtc2^LKO^* mice. mRNA levels (**b**) and protein levels (**c**) of Crtc2 in several key tissues showing the specificity of liver-specific depletion of *Crtc2* in 6h-fasted, 12 week-old mice under normal chow diet (NCD). **d.** Glucose tolerance test (GTT, left), pyruvate tolerance test (PTT, middle), and insulin tolerance test (ITT, right) showing effects of chronic depletion of hepatic *Crtc2* in 12 week-old mice under normal chow diet (NCD) on glucose metabolism and insulin signaling (n=5 mice per group). GTT and PTT was performed after 16 h-fasting, and ITT were performed after 6 h-fasting. **e.** 16 h-fasting body weight (left), and 6 h-fasting (fasted) blood glucose levels (right) from either *Crtc2^f/f^* mice or *Crtc2^LKO^* mice under NCD (n=5 mice per group). **f.** Effects of chronic depletion of hepatic *Crtc2* in 16 h-fasted mice under NCD on expression of hepatic genes (Q-PCR, n=5 mice per group). Data in **f** represent mean ± s.d. (*; P<0.05, **; P<0.01, t-test), and data in **d** and **e** represent mean ± s.e.m. (*; P<0.05, **; P<0.01, t-test).


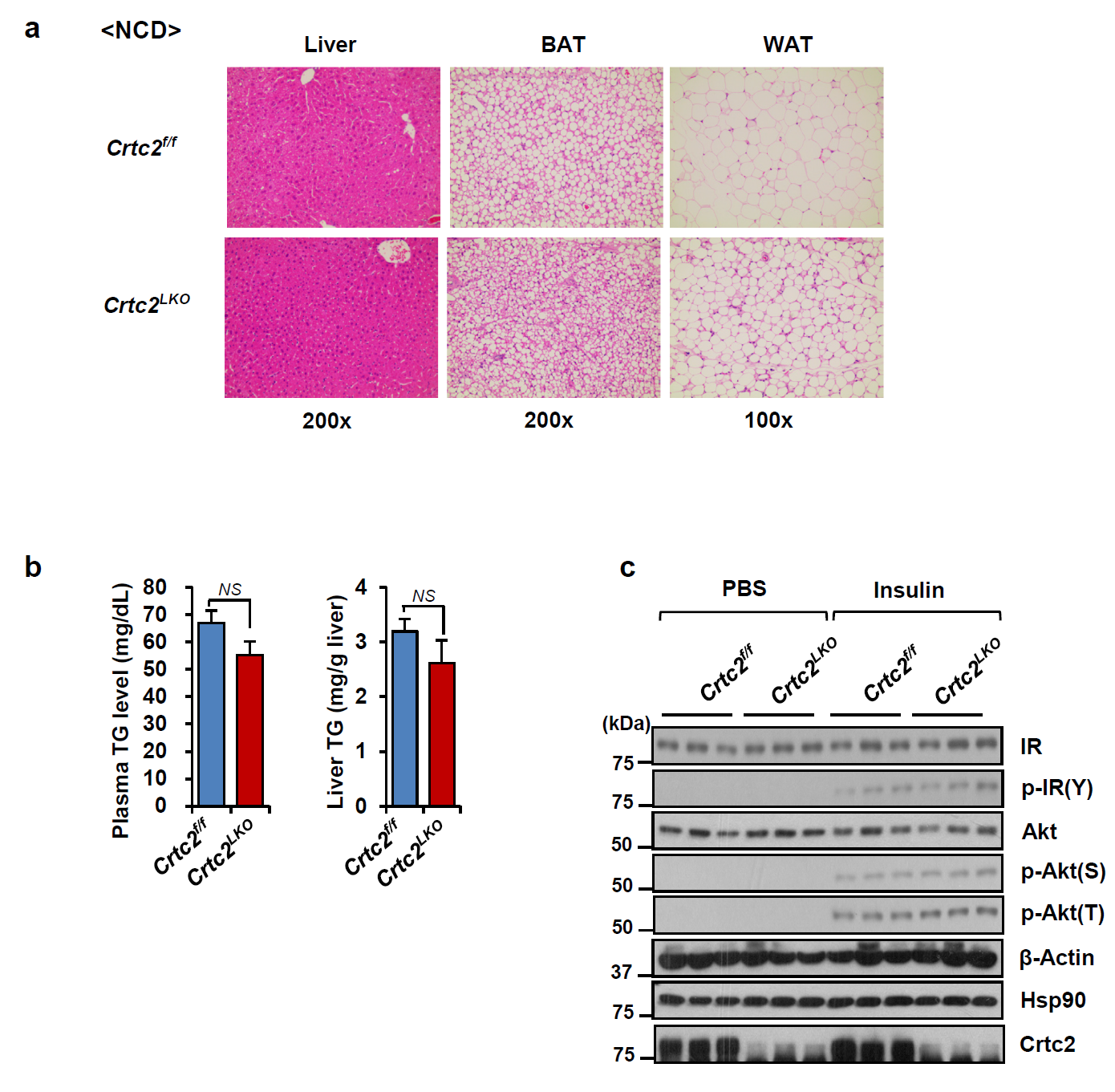


**Supplementary Figure 2. Effects of chronic depletion of hepatic *Crtc2* on lipid homeostasis and insulin signaling.**

**a.** Paraffin-embedded sections of subcutaneous liver (left), brown adipose tissues (BAT, middle), and visceral white adipose tissues (WAT, right) from 16 h-fasted, 17 week-old NCD-fed *Crtc2^f/f^* mice or *Crtc2^LKO^* mice were stained with H&E. Data represent 3 independent experiments (n=3 mice per group). **b.** Plasma triglycerides (TG) levels (left) and hepatic TG levels (right) from either *Crtc2^f/f^* mice or *Crtc2^LKO^* mice as in Fig. S2**a** (n=6 mice per group). **c.** Effects of chronic depletion of hepatic *Crtc2* in 9 week-old, 6 h-fasted mice under NCD on hepatic proteins in the insulin signaling pathway after a bolus of insulin injection (0.1 unit/mice) (n=4~6 mice per group). Data in **b** represent mean ± s.e.m. (NS; not significant, t-test).


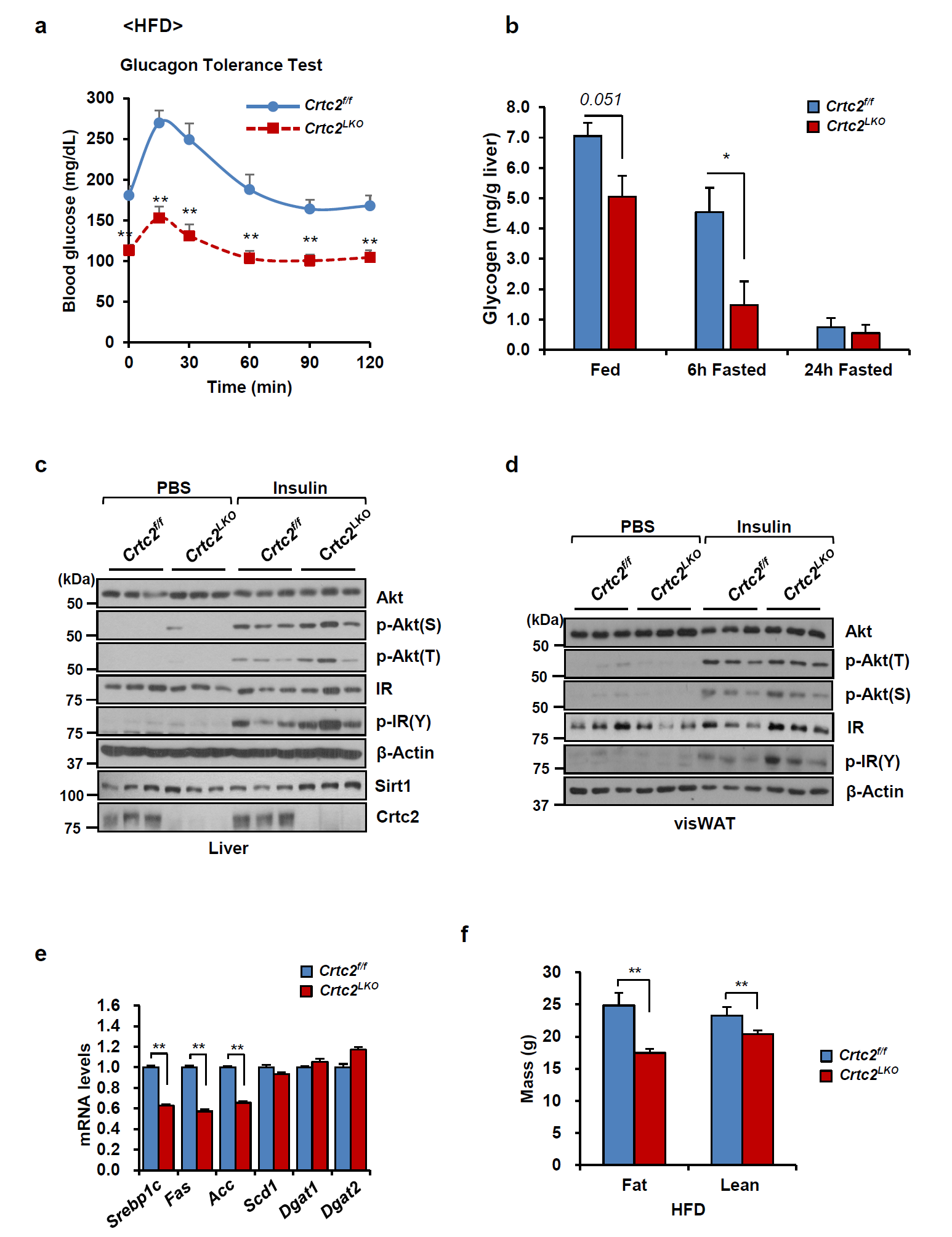


**Supplementary Figure 3. Effects of chronic depletion of hepatic *Crtc2* on glucagon response, insulin signaling, and lipid metabolism in mice.**

**a.** Glucagon tolerance test showing effects of chronic depletion of hepatic *Crtc2* in 6 h-fasted mice under high fat diet (HFD) for 6 weeks on glucagon-responsive changes in plasma glucose levels (n=7~8 mice per group). **b**. Hepatic glycogen levels from ad libitum (fed), 6 h-fasted and 24 h-fasted *Crtc2^f/f^* mice or *Crtc2^LKO^* mice under HFD for 6 weeks (n=5~7 mice per group). **c-d.** Effects of chronic depletion of hepatic *Crtc2* in mice under 9 week-HFD on insulin signaling in the liver (**c**) or visceral WAT (**d**) after a bolus of insulin injection (0.1 unit/mice) (n=5~6 mice per group). **e.** Effects of chronic depletion of hepatic *Crtc2* in ad libitum-mice under 9 week-HFD on lipogenic genes. **f.** Effects of chronic depletion of hepatic *Crtc2* in mice in the fat body mass and the lean body mass (n=5~6 mice per group) under 23 week-HFD (n=3 for *Crtc2^f/f^* mice and n=7 for *Crtc2^LKO^* mice). Data in **e** represent mean ± s.d. (**; P<0.01, t-test), and data in **a**, **b** and **f** represent mean ± s.e.m.(*; P<0.05, **; P<0.01, t-test).


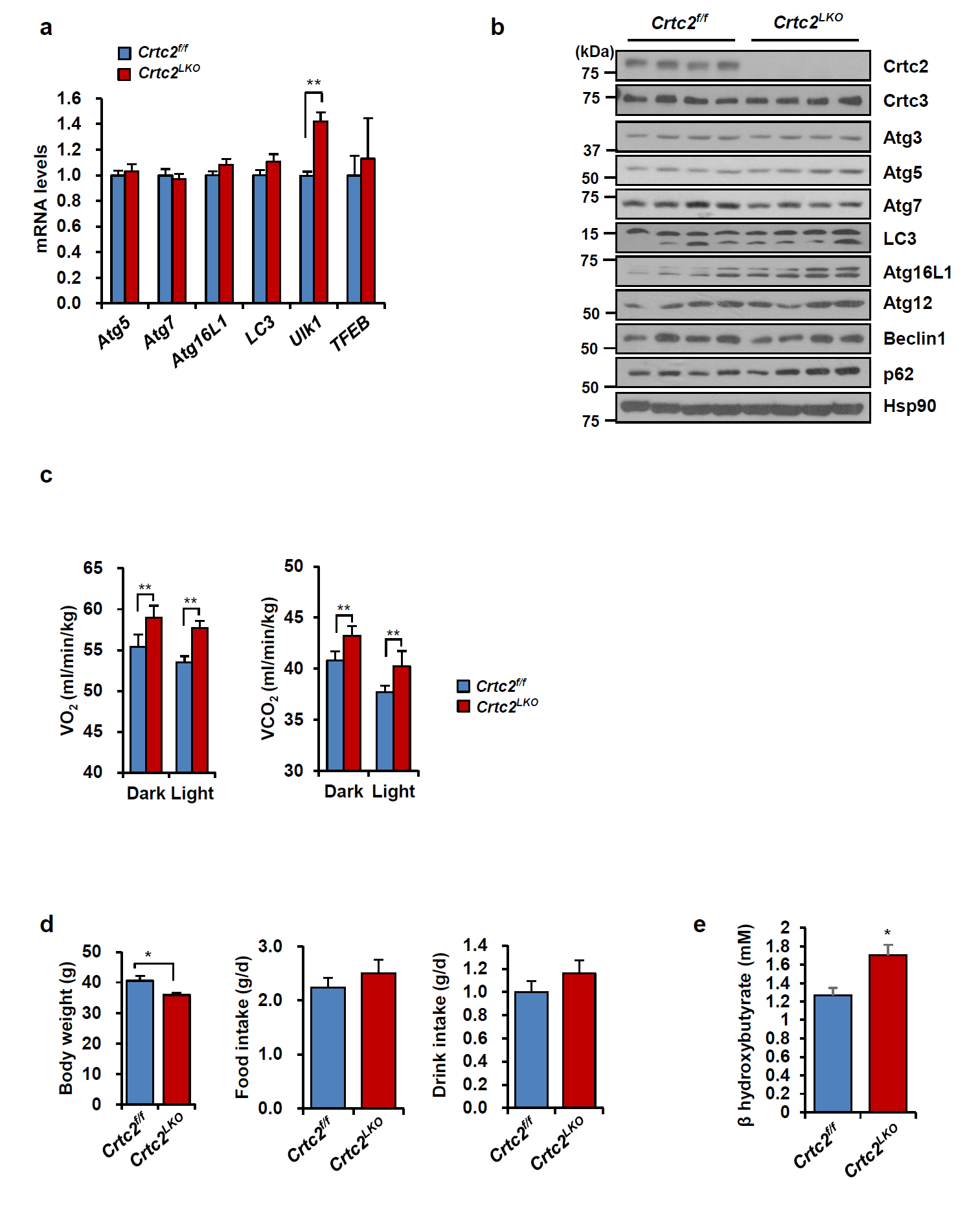


**Supplementary Figure 4. Effects of chronic depletion of hepatic *Crtc2* on autophagy and energy homeostasis in mice.**

**a.** Effects of chronic depletion of hepatic *Crtc2* in 16 h-fasted mice under 9 week-HFD on genes involved in the autophagy. **b.** Effects of chronic depletion of hepatic *Crtc2* in 16 h-fasted mice under 9 week-HFD on proteins involved in the autophagy. **c**-**d.** Oxygen consumption (VO_2_, **c**, top), carbon dioxide production (VCO_2_, **c**, bottom), body weight (**d**, left), daily food consumption (**d**, middle), and daily water consumption (**d**, right) were measured from 7 week-HFD-fed *Crtc2^f/f^* mice or *Crtc2^LKO^* mice by using metabolic cage (n=10 mice per group). **e.** Effects of chronic depletion of hepatic *Crtc2* on plasma ketone bodies in 16 h fasted-mice under 23 week-HFD (n=3 for *Crtc2^f/f^* mice and n=7 *for Crtc2^LKO^* mice). Data in **a** represent mean ± s.d. (**; P<0.01, t-test), and data in **c**- **e** represent mean ± s.e.m. (*; P<0.05, **; P<0.01, t-test).


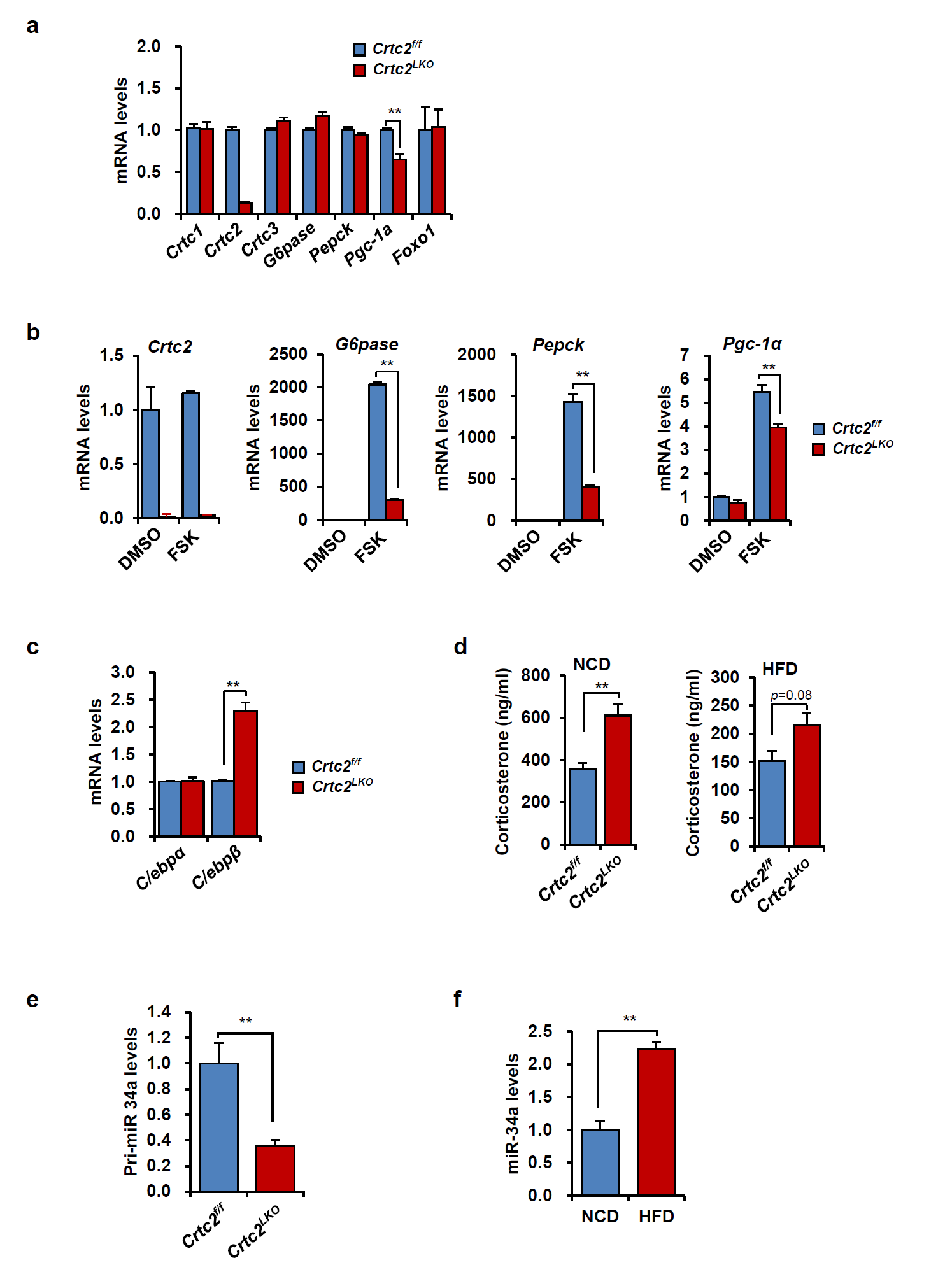


**Supplementary Figure 5. Effects of Creb/Crtc2 on hepatic gluconeogenesis.**

**a.** Effects of chronic depletion of hepatic *Crtc2* on *Crtcs* and gluconeogenic genes in 16 h-fasted mice under 9 week-HFD (Q-PCR, n=4 mice per group). **b.** Effects of *Crtc2* knockout on gluconeogenic genes in primary hepatocytes in the absence or in the presence of cAMP agonist forskolin (Q-PCR, n=3 sets of cells per group). **c.** Effects of chronic depletion of hepatic *Crtc2* on expression of *C/ebp* alpha and beta isoforms in 16 h-fasted mice under 9 week-HFD (Q-PCR, n=4 mice per group). **d.** Effects of chronic depletion of hepatic *Crtc2* on plasma corticosterone levels in 16 h-fasted mice under NCD (left) or HFD (9-week) (right) (n=4 mice per group). **e.** Effects of chronic depletion of hepatic *Crtc2* on primary miR-34a expression in 16 h-fasted mice under 9 week-HFD (n=4 mice per group). **f.** Effects of 8 week-HFD on hepatic miR-34a expression in mice under ad libitum (n=4 mice per group). Data in **a**-**c**, **e** and **f** represent mean ± s.d. (**; P<0.01, t-test), and data in **d** represent mean ± s.e.m. **; P<0.01, t-test).


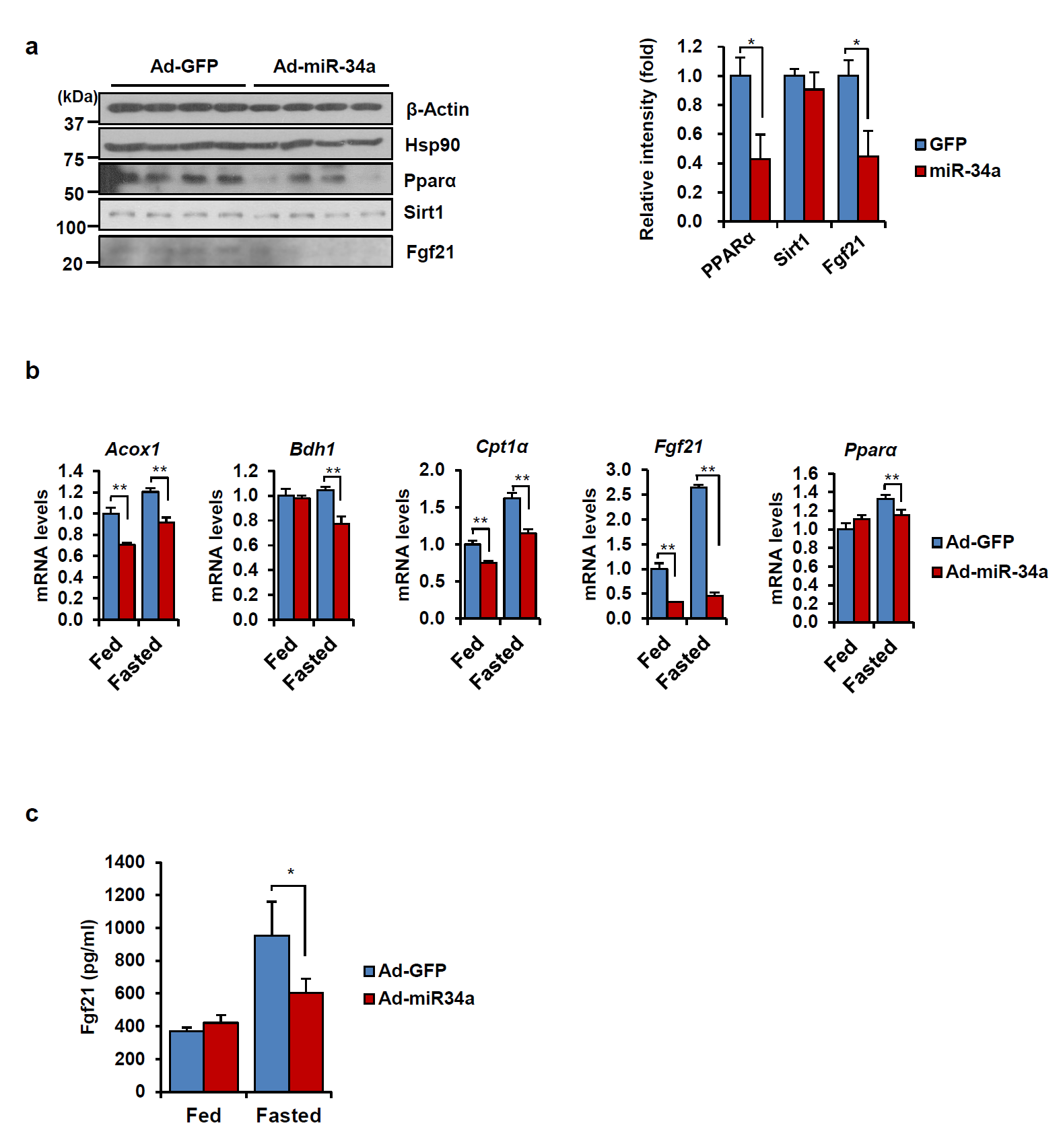
 **Supplementary Figure 6. Effects of miR-34a on hepatic expression of Pparα target genes.**

**a-c.** Effects of hepatic expression of miR-34a on Fgf21. Hepatic protein levels and their quantitation under 16 h-fasting (**a**), hepatic expression of *Pparα* and its target genes under ad libitum and 16 h-fasting (**b**), and ad libitum and 16 h-fasting plasma Fgf21 levels (**c**) from 8 week-old C57BL/6 mice that were infected with Ad-GFP or Ad-miR-34a adenovirus for 5 days (n=5 mice per group). Data in **a** and **b** represent mean ± s.d. (*; *P*<0.05, **; *P*<0.01, t-test), and data in **c** represent mean ± s.e.m. (*; *P*<0.05, Tukey-Kramer Multiple Comparisons).


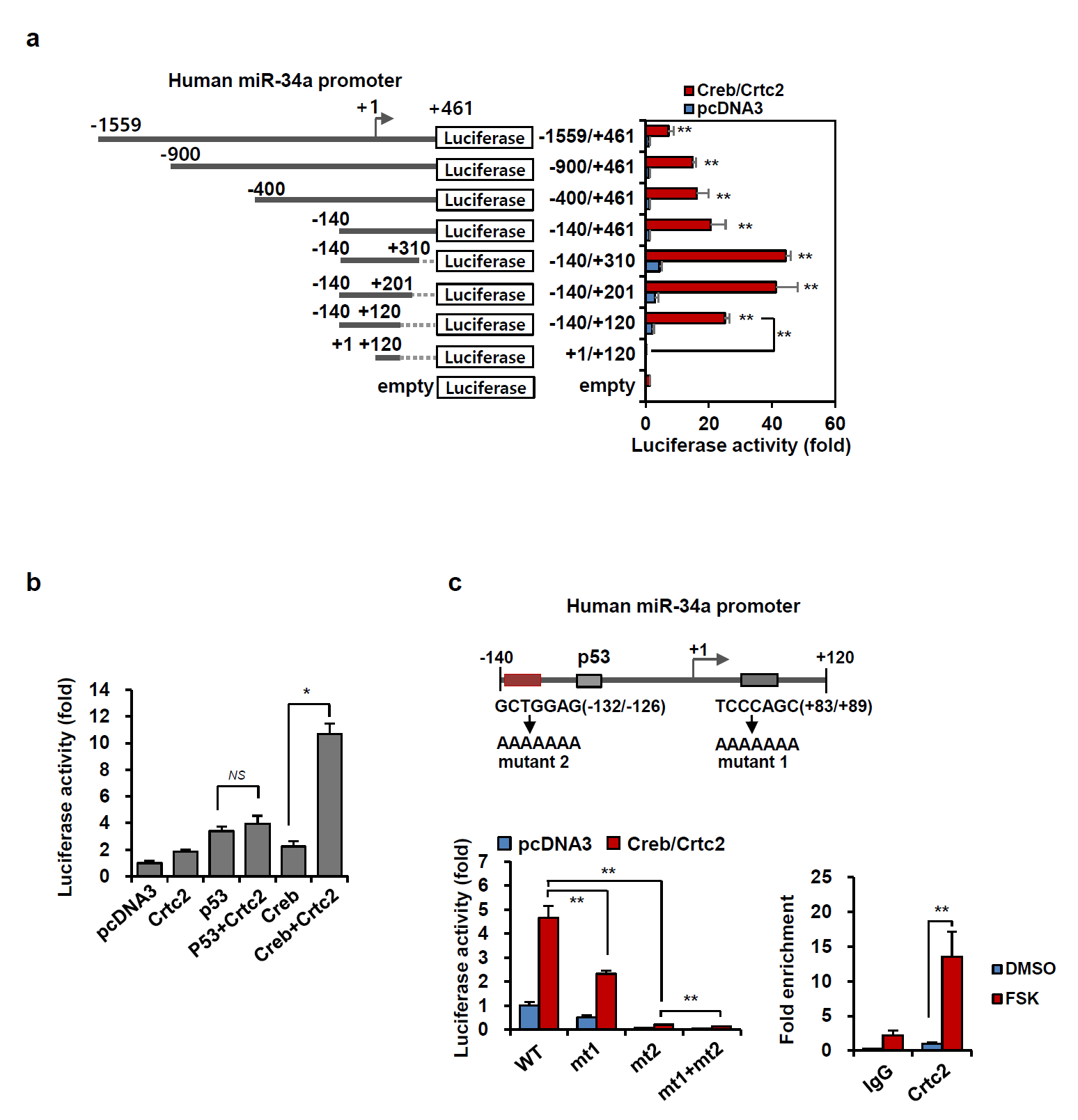
 **Supplementary Figure 7. Effects of Creb/Crtc2 on expression of human miR-34a.**

**a-b.** Luciferase reporter assay was performed in 293T cells to determine the effects of p53 or Creb/Crtc2 on miR-34a promoter activity (**a**). 5’- and 3’-deletion analysis was performed to map the putative Creb/Crtc2 response element on the promoter of human miR-34a (**b**). N=3 independent experiments in triplicate. **c.** Location of putative CREs on the human miR-34a promoter (left), the effects of CRE mutations on the human miR-34a promoter activity (middle), and the chromatin immunoprecipitation assay showing the occupancy of Crtc2 over human miR-34a promoter (right) were shown. N=3 independent experiments in triplicate. Data in **a**-**c** represent mean ± s.d. (*; P<0.05, **; P<0.01, Tukey-Kramer Multiple Comparisons).


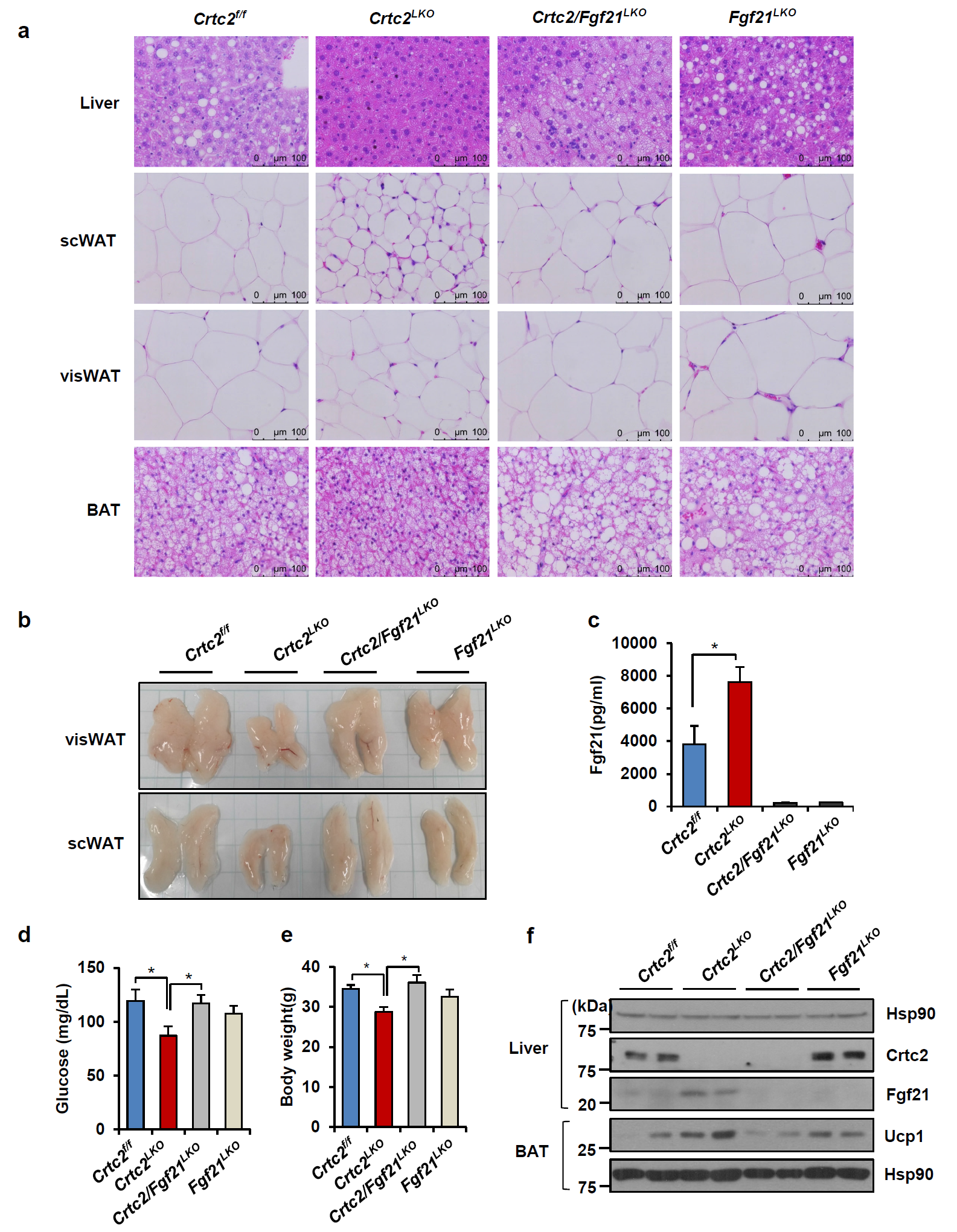


**Supplementary Figure 8.** **Depletion of *Fgf21* reverted the effect of liver-specific knockout of *Crtc2* on energy metabolism in DIO mice.**

**a.** Paraffin-embedded sections of liver tissues, visceral white adipose tissues (visWAT), subcutaneous white adipose tissues (scWAT), and brown adipose tissues (BAT) from 16 h-fasted, 11 week-HFD-fed *Crtc2^f/f^* mice, *Crtc2^LKO^* mice, *Crtc2/Fgf21^LKO^* mice, or *Fgf21^LKO^* mice were stained with hematoxylin and eosin (H&E) (n=4 mice per group). **b**. Fat size of each group of mice was shown. **c**. Effects of chronic liver-specific depletion of *Crtc2* and/or *Fgf21* in 16 h-fasted mice under 11 week-HFD on plasma Fgf21 levels (n=5~7 mice per group). **d**. 16 h-fasting blood glucose levels from *Crtc2^f/f^* mice, *Crtc2^LKO^* mice, *Crtc2/Fgf21^LKO^* mice, or *Fgf21^LKO^* mice under HFD for 11 weeks (n=5~7 mice per group). **e.** 16 h**-**fasting body weight from *Crtc2^f/f^* mice, *Crtc2^LKO^* mice, *Crtc2/Fgf21^LKO^* mice, or *Fgf21^LKO^* mice under HFD for 11 weeks (n=5~7 mice per group). **f.** Effects of hepatic *Crtc2* and/or *Fgf21* knockout on Fgf21 protein levels in the liver, and Ucp1 protein levels in the BAT from 16 h-fasted, 11 week-HFD fed mice. Data in represent mean ± s.e.m. (*; P<0.05, Tukey-Kramer Multiple Comparisons).


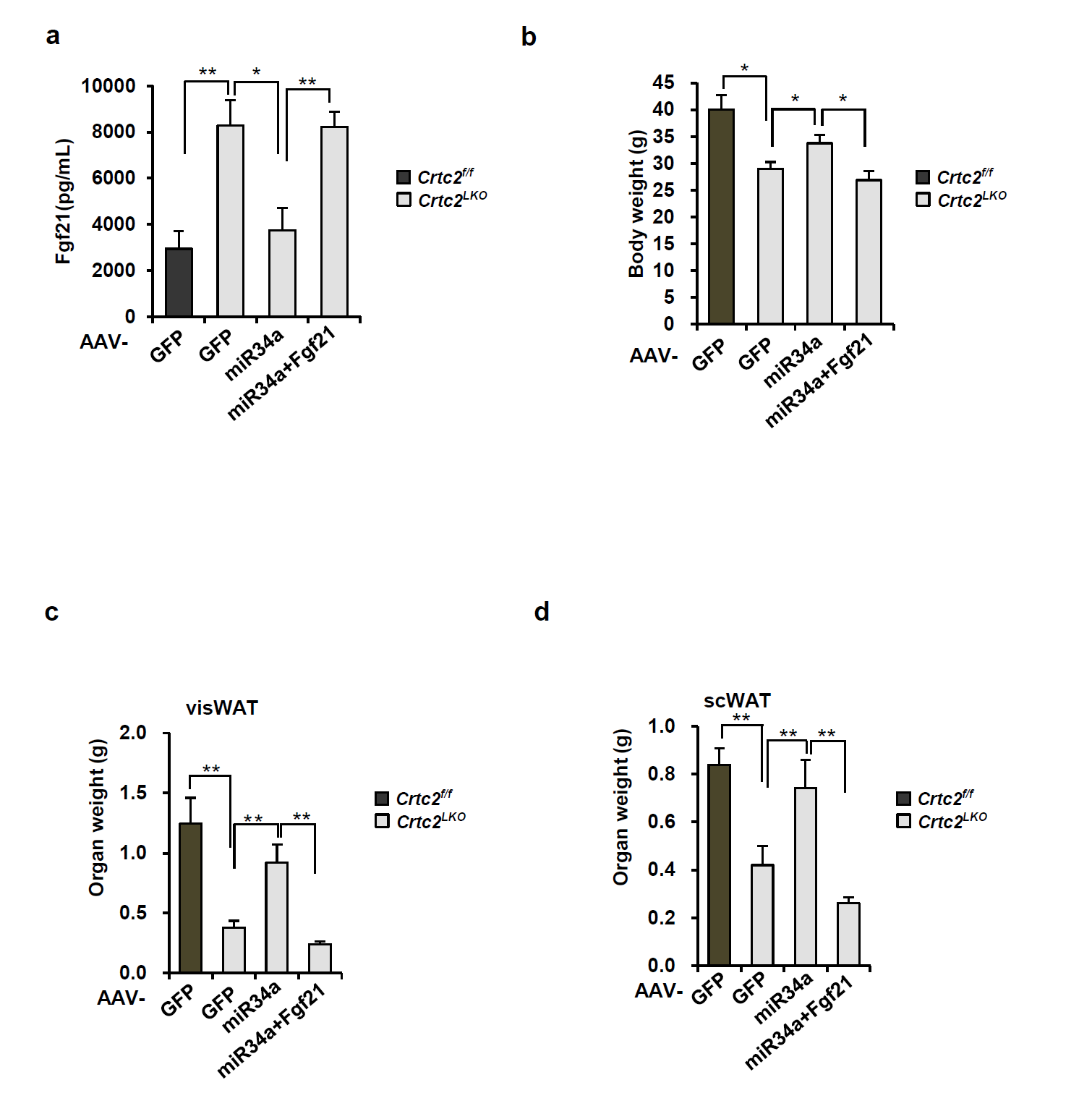
 **Supplementary Figure 9. Effect of miR-34a on adiposity is reversed by co-expression of Fgf21 in *Crtc2* liver-specific knockout mice**.

**a**-**d**. Effects of miR-34a and/or Fgf21 on *Crtc2^LKO^* mice under 9 week-HFD (n=5 mice per group). Plasma Fgf21 levels (**a**), body weight (**b**), vWAT weight (**c**), and scWAT weight (**d**) from mice of each genotype were shown. Data represent mean ± s.e.m. (*; P<0.05, **; P<0.01, Tukey-Kramer Multiple Comparisons).


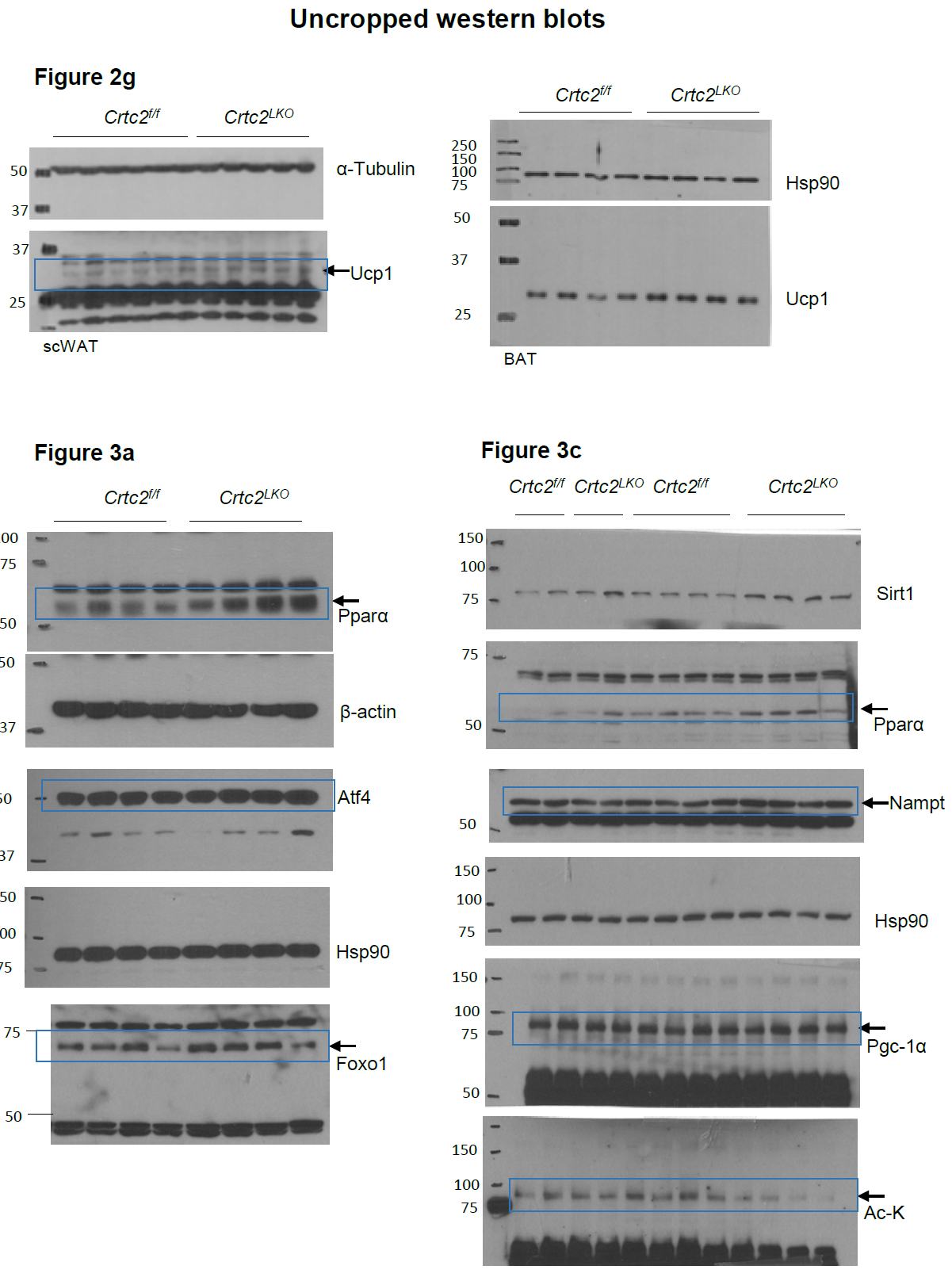
 **Supplementary Figure 10. Uncropped western blot images for Figure 2g, 3a, and 3c**


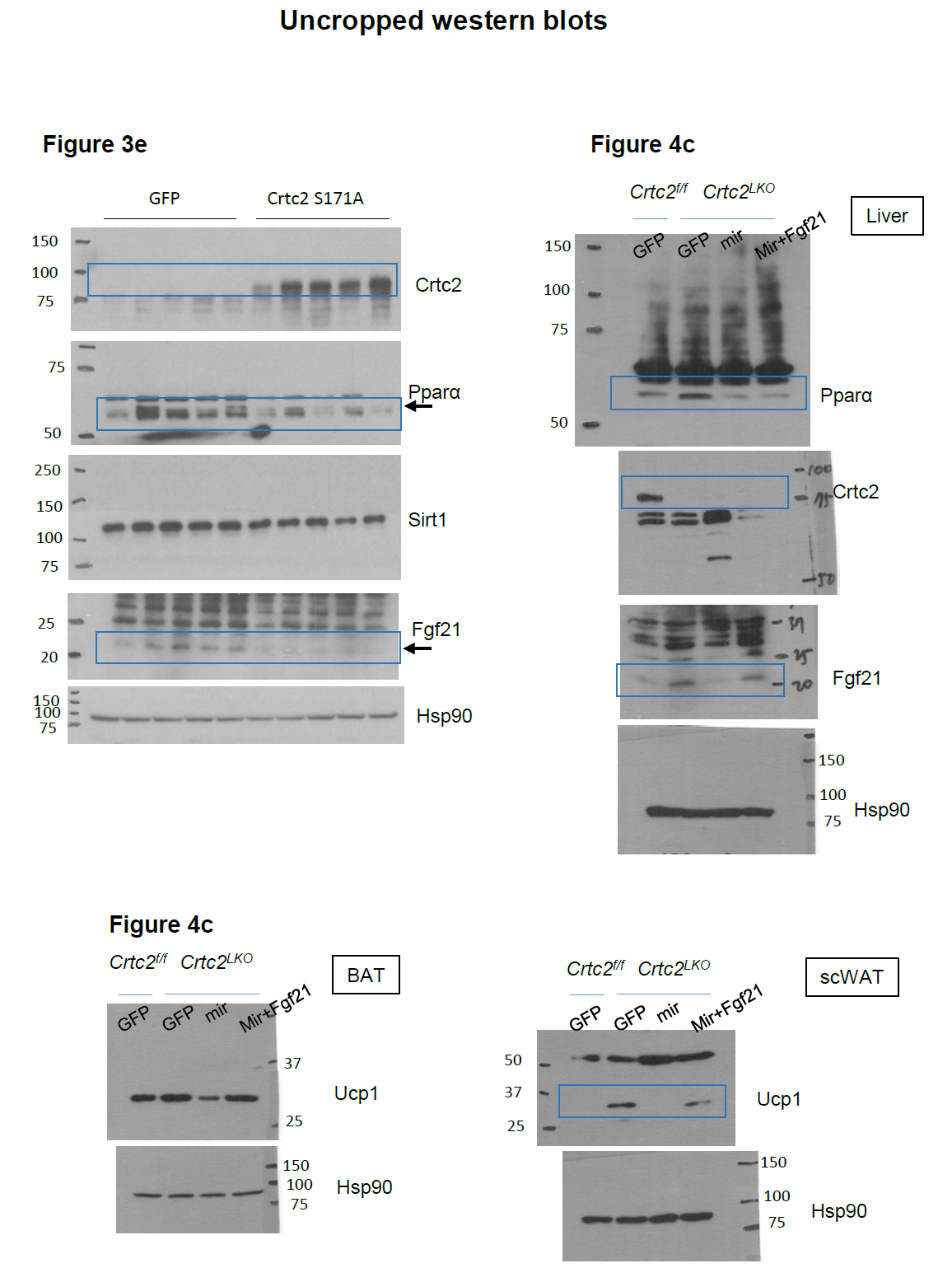


**Supplementary Figure 11. Uncropped western blot images for Figure 3e and 4c**


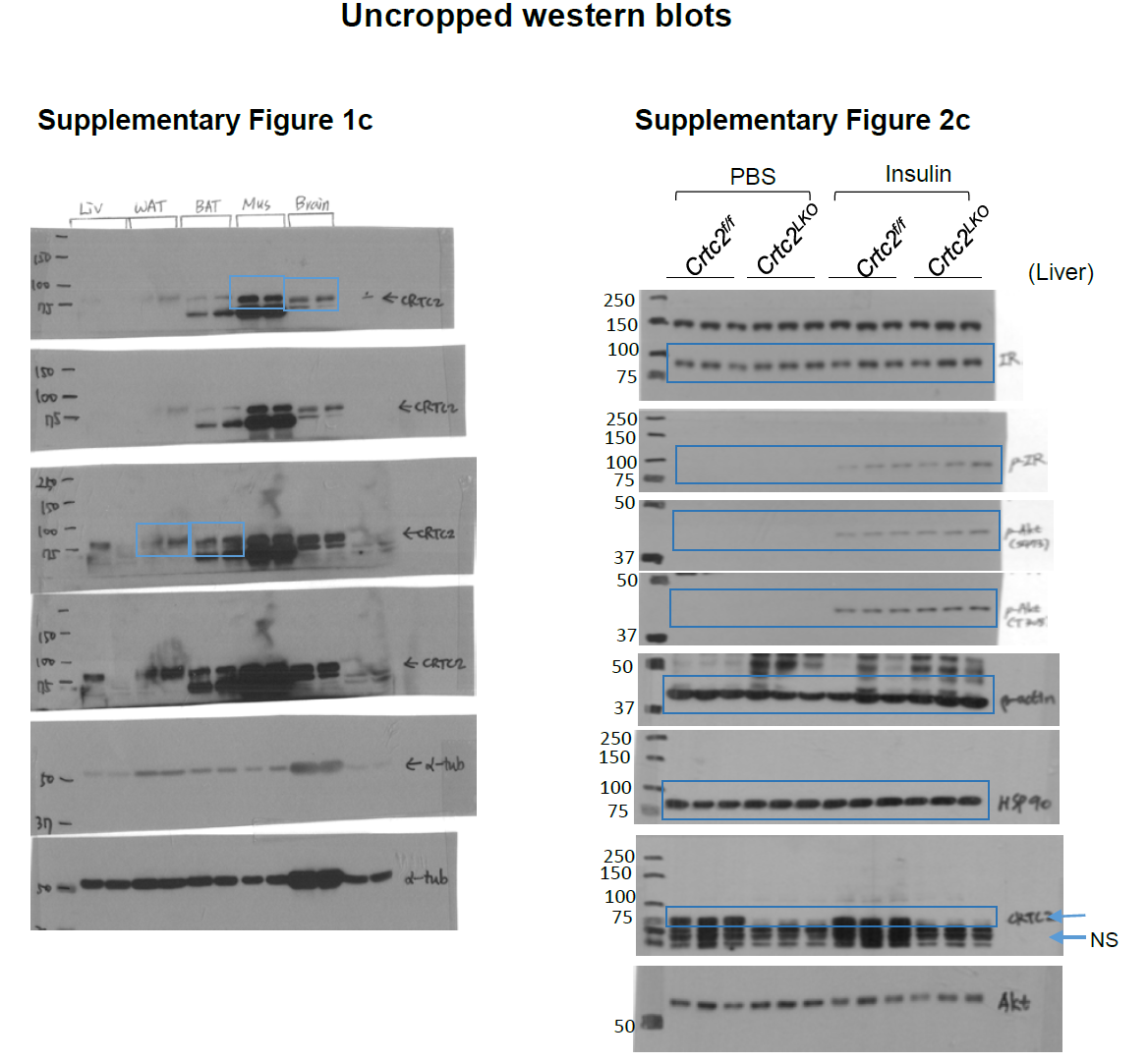
 **Supplementary Figure 12. Uncropped western blot images for Supplementary Figure 1c and 2c**


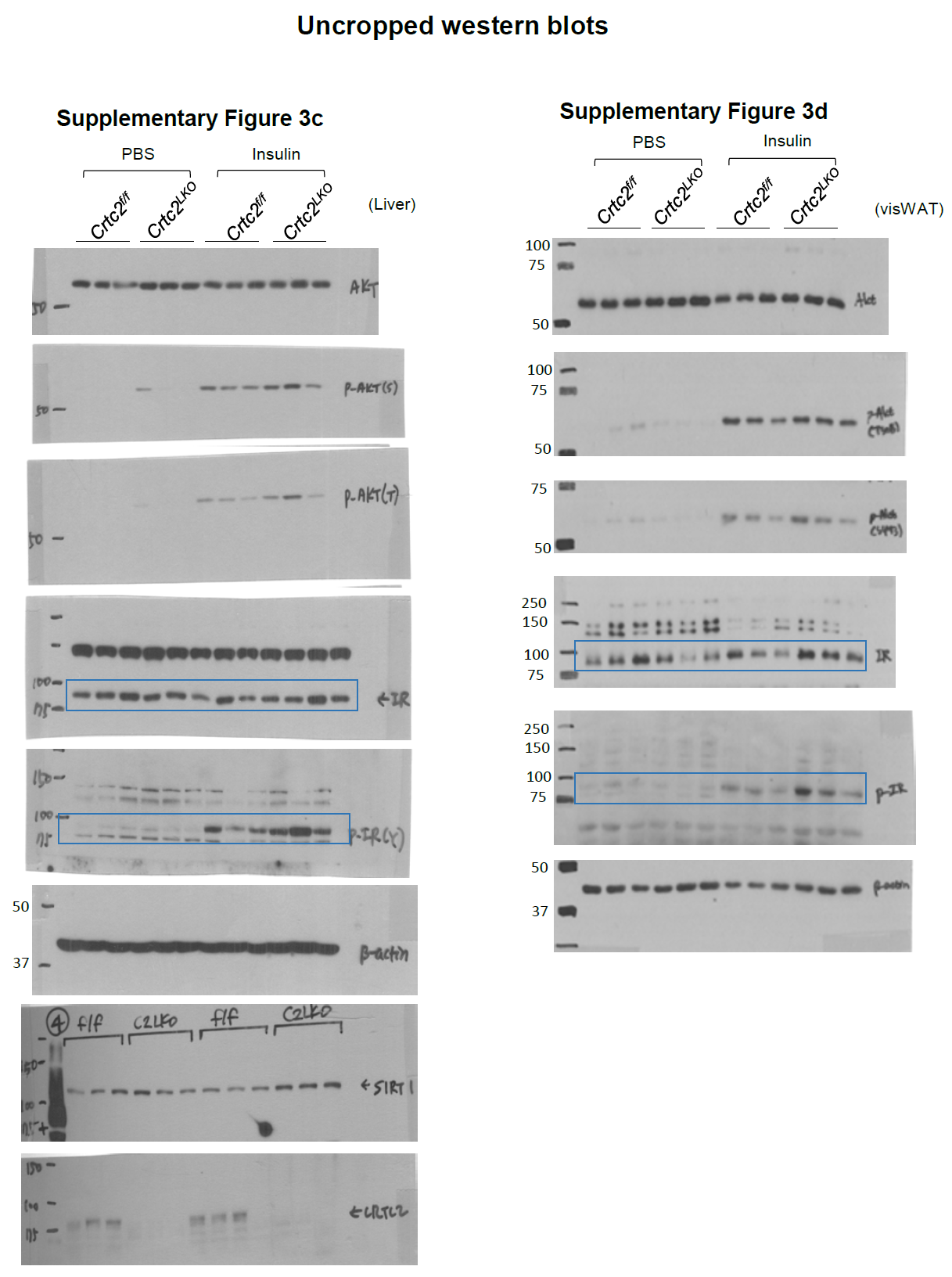
 **Supplementary Figure 13. Uncropped western blot images for Supplementary Figure 3c and 3d**


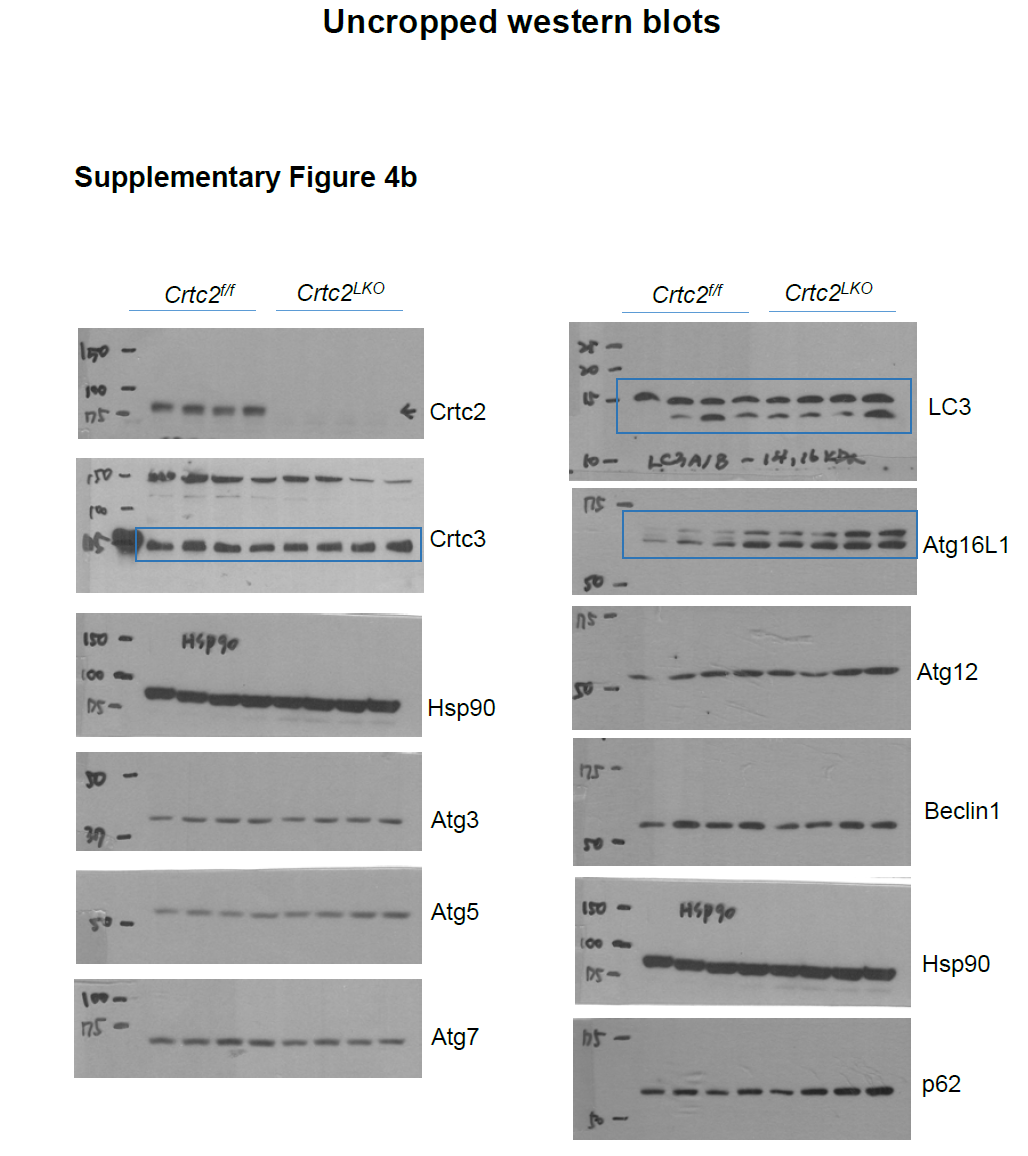
 **Supplementary Figure 14. Uncropped western blot images for Supplementary Figure 4b**


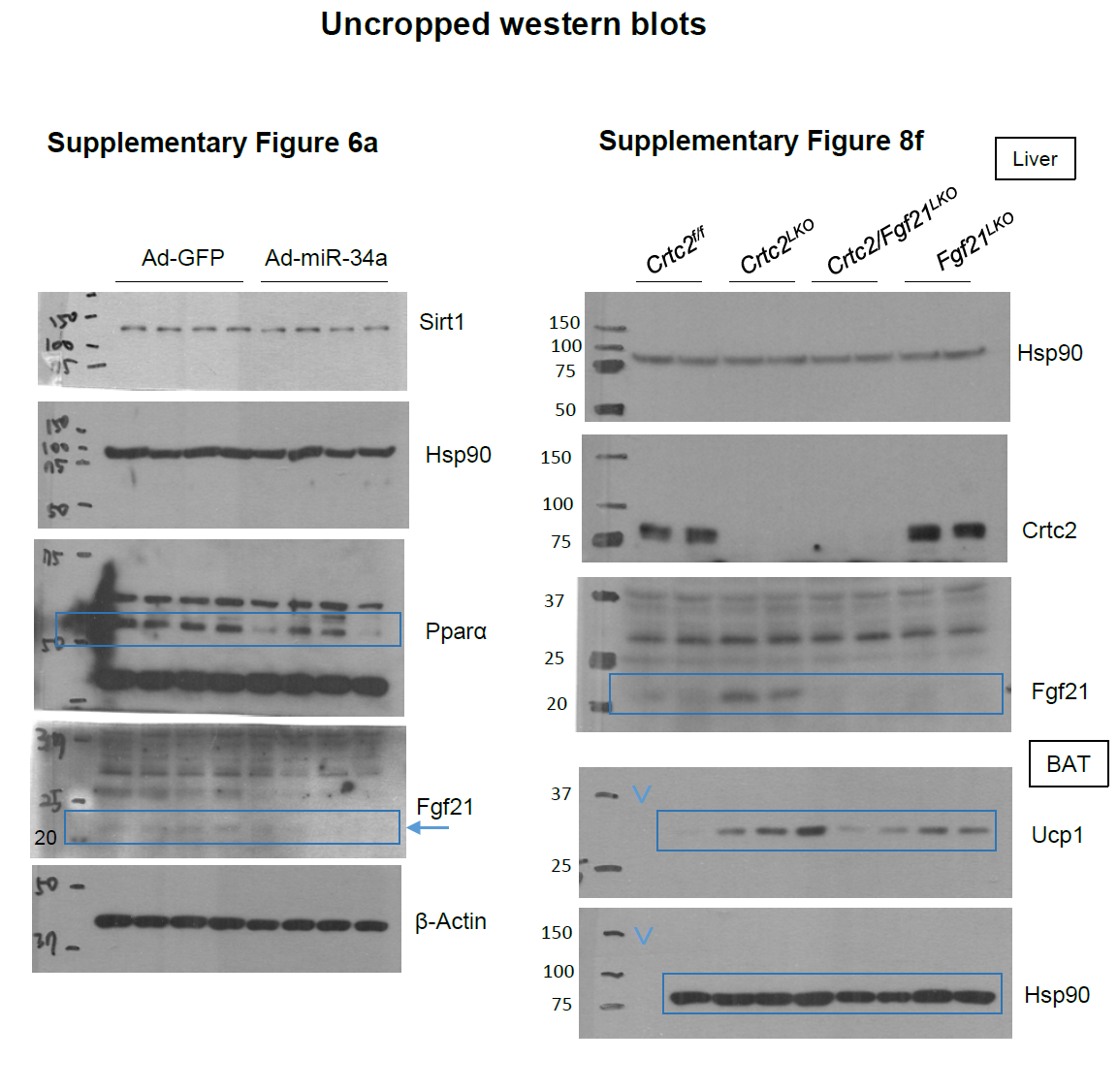


**Supplementary Figure 15. Uncropped western blot images for Supplementary Figure 6a and 8f**


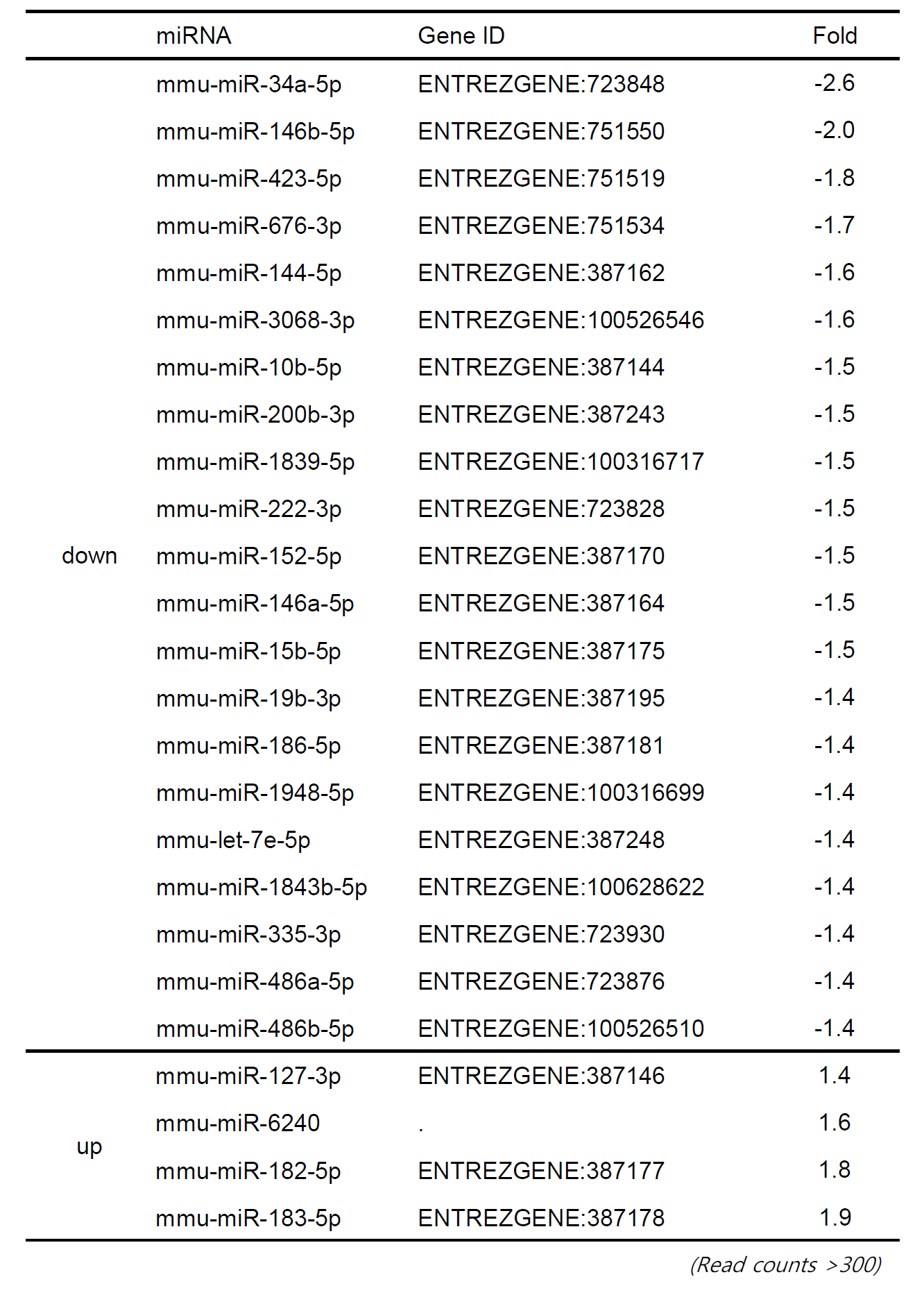
 **Supplementary Table 1. A representative list of miRNA that were affected by hepatic depletion of *Crtc2* in mice.**

Effects of chronic depletion of hepatic *Crtc2* in 16 h-fasted mice under 9 week-HFD on miR-34 were analyzed by small RNA sequencing analysis (n=3 per group).


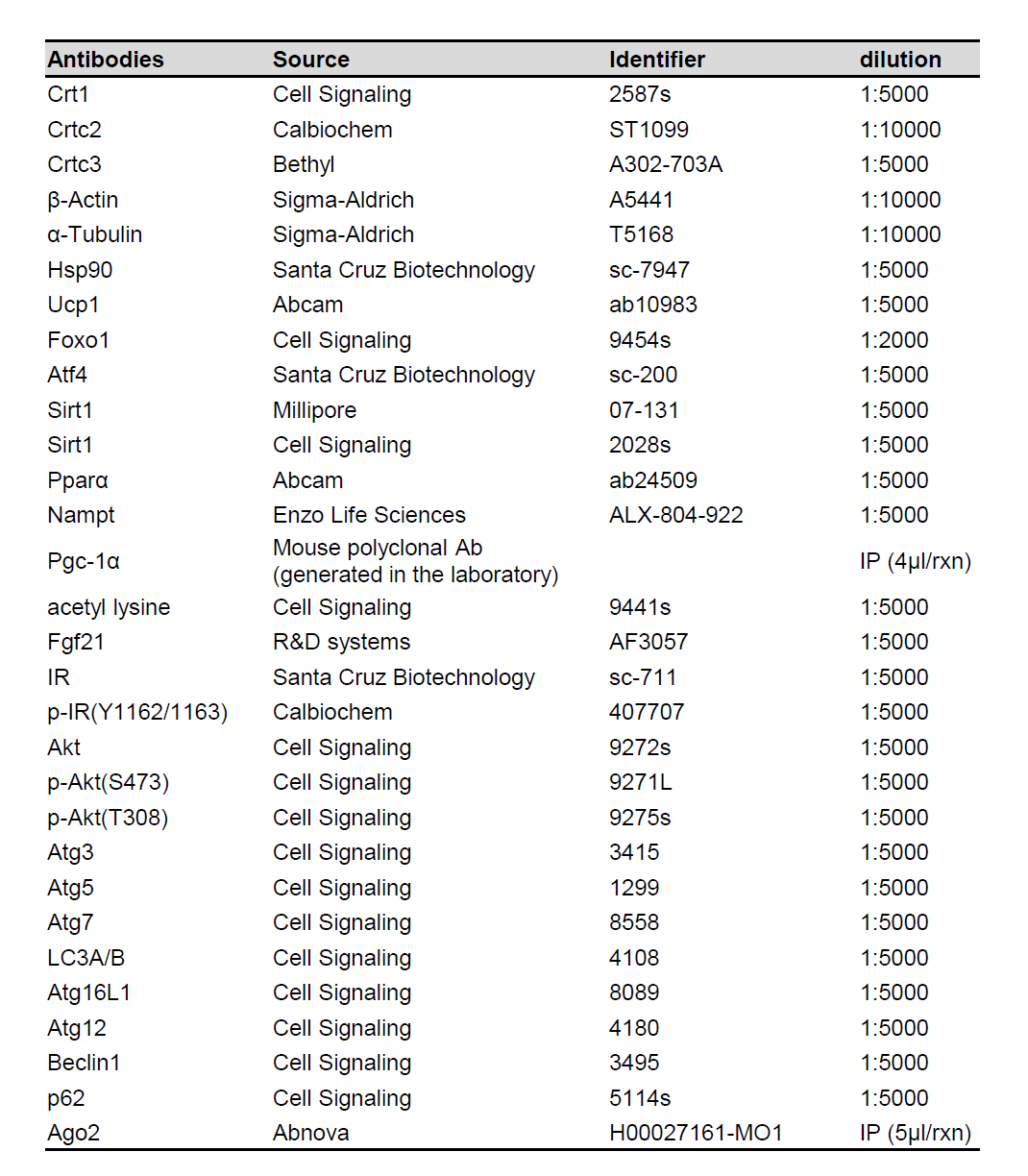
**Supplementary Table 2. Information for antibodies utilized in the western blot analysis and immunoprecipitation**


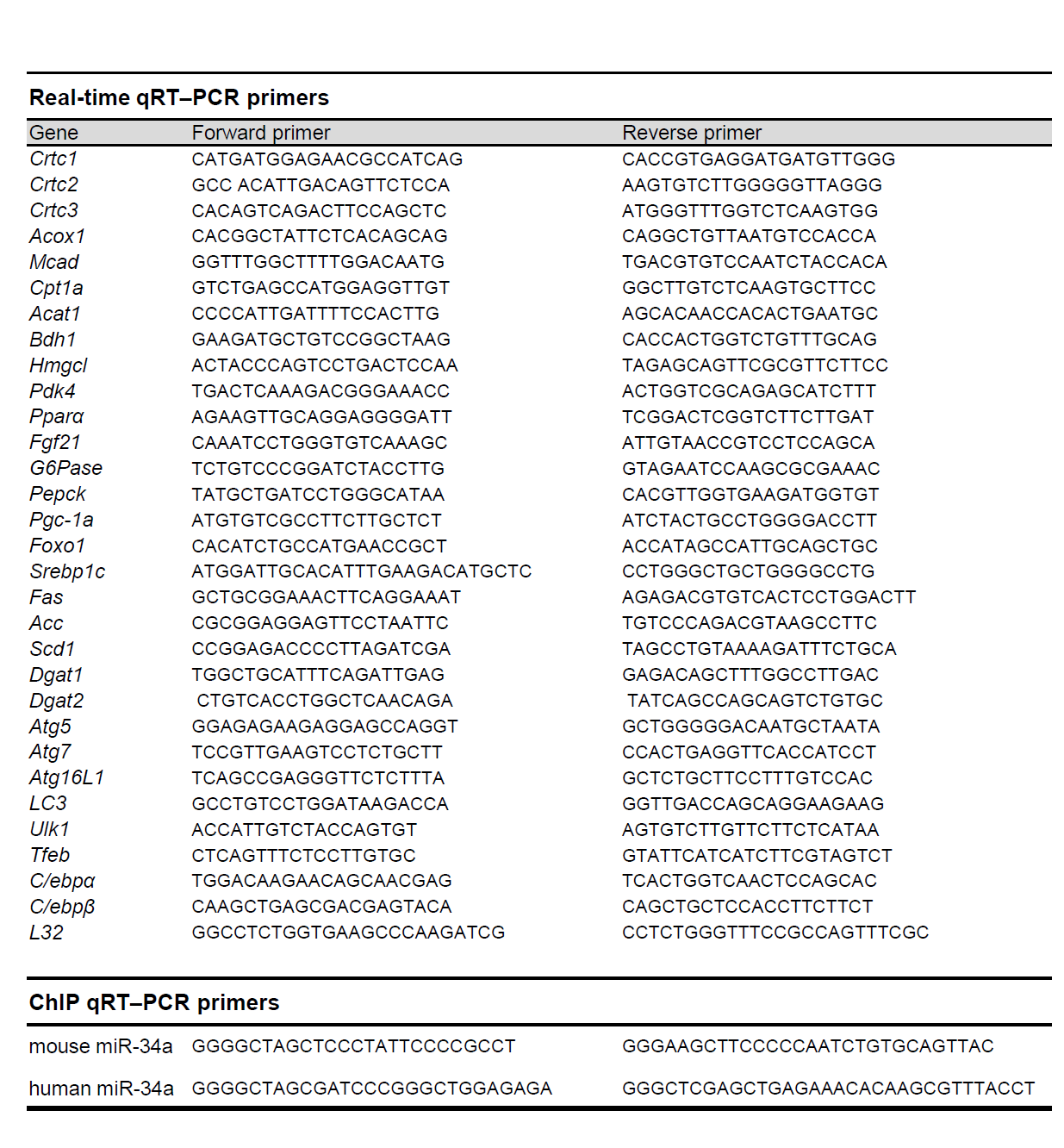
 **Supplementary Table 3. Sequence information for qRT-PCR primers**
